# Right main bronchus rupture due to blunt chest trauma

**DOI:** 10.1002/ccr3.1953

**Published:** 2018-12-04

**Authors:** Sharfuddin Chowdhury, John Griniatsos

**Affiliations:** ^1^ Trauma Unit, King Saud Medical City Riyadh Kingdom of Saudi Arabia; ^2^ 1st Department of Surgery National and Kapodistrian University of Athens Athens Greece

**Keywords:** blunt trauma, chest trauma, main bronchus rupture, tracheobronchial injury

## Abstract

Bronchial rupture following major blunt chest trauma should be suspected in every case of massive and persistent air leak through the intercostal drain tube. Chest radiogram offers indirect signs, while chest CT scan demonstrates specific signs highly suggestive for this extremely rare tracheobronchial injury. Bedside bronchoscopy confirms the diagnosis.

## 1 CLINICAL IMAGE—CASE PRESENTATION

**Figure 1:**
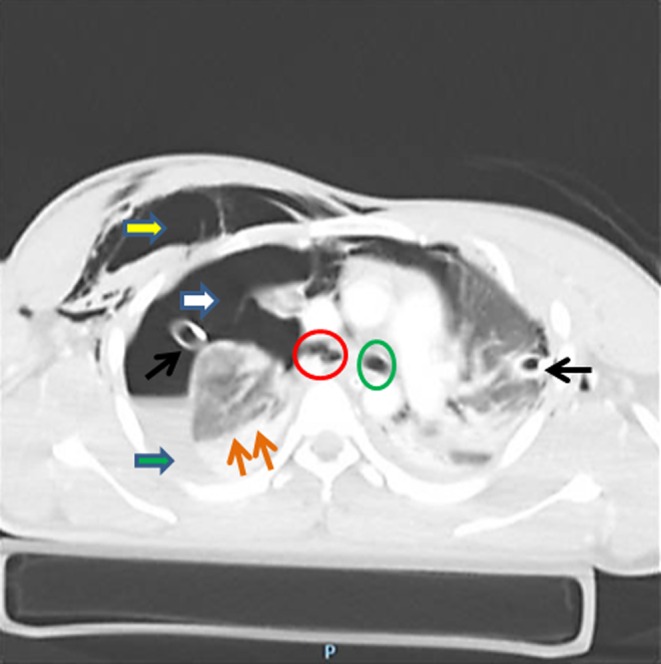
Axial plane view of the CT chest on lung window shows right‐sided subcutaneous emphysema (yellow arrow), right pneumothorax (white arrow), right hemothorax (green arrow), “fallen lung” sign (double brown arrows), and the presence of bilateral chest tubes (black arrows). The left main bronchus silhouette is intact (green circle), while the outline of the right main bronchus is disrupted (red circle). The above radiological findings are highly suggestive of right main bronchus rupture. A bedside bronchoscopy confirmed the diagnosis

A 26‐year‐old male worker sustained bilateral hemopneumothorax after a wall collapsed on his chest. On admission, auscultation of thorax disclosed diminished air entry bilaterally. He was managed initially with bilateral intercostal drains. Massive air leak and failure for lung expansion were noticed on the right side of the chest. After initial resuscitation, he underwent chest CT scan as a part of the trauma series, which was highly suggestive for an extremely rare tracheobronchial injury (Figure 1 **)**.

## 2 ANSWER—DISCUSSION

The fact that right‐sided bronchial injuries occur more frequently probably related to the shorter length of the right main bronchus, and to the heavier right lung which may cause more traction on the right bronchus and because the left bronchus is relatively protected by its longer mediastinal course and the surrounding peribronchial tissues.^1^

Chest radiogram offers indirect signs, while chest CT scan demonstrates the “fallen lung” sign; since the air leaks through the rupture site leading to lung collapse, the intact pulmonary vessels are unable to sustain it and the lung drops toward the diaphragm, hence in a supine patient the collapsed lung occupies the lateral and posterior part of the affected hemithorax.^2^

## CONFLICT OF INTEREST

None declared.

## AUTHORS’ CONTRIBUTION

SC: chose the case, made useful comments and corrections, and gave the final approval. JG: wrote the article. Both authors have read and approved the final version for publication.
